# A novel anti-p21Ras scFv antibody reacting specifically with human tumour cell lines and primary tumour tissues

**DOI:** 10.1186/s12885-016-2168-6

**Published:** 2016-02-20

**Authors:** Ju-Lun Yang, Du-Xian Liu, Shi-Jian Zhen, Yun-Gang Zhou, Dai-Jun Zhang, Li-Ying Yang, Hao-Bing Chen, Qiang Feng

**Affiliations:** Department of Pathology, Kunming General Hospital/Kunming Medical University, Kunming, 650032 Yunnan Province China; Department of Molecular Biology, Kunming General Hospital/Kunming Medical University, Kunming, 650032 Yunnan Province China

**Keywords:** p21Ras, scFv, Tumour, Immunoreactivity, Monoclonal antibody

## Abstract

**Background:**

The *ras* genes play an important role in the development and progression of human tumours. Neutralizing Ras proteins in the cytoplasm could be an effective approach to blocking *ras* signalling. In this study, we prepared anti-p21Ras single chain fragment variable antibody (scFv) and investigated its immunoreactivity with human tumours.

**Methods:**

The coding sequences of *H-ras*, *K-ras*, and *N-ras* were separately ligated into the vector pET-28a(+). Then, recombinant expressing plasmids were induced by IPTG for p21Ras expression in *E. coli*. Hybridoma cell lines producing anti-p21Ras monoclonal antibodies were isolated using wildtype p21Ras proteins as immunogens. Anti-p21Ras scFv antibody was prepared from the hybridoma by the phage scFv display method. The immunoreactivity of the anti-p21Ras monoclonal antibody and the scFv antibody was identified by ELISA and immunocytochemistry.

**Results:**

We prokaryotically expressed wildtype H-p21Ras, K-p21Ras and N-p21Ras and generated the hybridoma cell line KGH-R1, producing anti-p21Ras monoclonal antibodies. It was demonstrated that KGH-R1 monoclonal antibody could recognize wildtype and mutated H-p21Ras, K-p21Ras and N-p21Ras in human tumour cell lines.

In all 14 types of primary human cancer tissues tested, the monoclonal antibody presented strong immunoreactivity but showed weak or negative immunoreactivity in the corresponding normal tissues. Subsequently, we prepared anti-p21Ras scFv from hybridoma KGH-R1, which showed the same immunoreactivity as the original monoclonal antibody. Sequence analysis demonstrated that the nucleotides and amino acids of the scFv exhibited an approximately 50 % difference from the anti-p21Ras scFv reported previously.

**Conclusions:**

This study presents a novel anti-p21Ras scFv antibody. Our data suggest that the scFv may be useful for ras signalling blockage and may be a potential therapeutic antibody for ras-derived tumours.

**Electronic supplementary material:**

The online version of this article (doi:10.1186/s12885-016-2168-6) contains supplementary material, which is available to authorized users.

## Background

Because of the important role of *ras* in carcinogenesis and progression, the ras signalling pathway has attracted considerable attention as a target for anticancer therapy. The *ras* gene product, p21Ras, is a monomeric membrane-localized G protein of 21 kD, which functions as a molecular switch converting signals from the cell membrane to the nucleus and linking receptor and nonreceptor tyrosine kinase activation to downstream cytoplasmic or nuclear events. The biological effects of p21Ras depend on its biochemical properties of being a small GTP-binding protein and on its correct cellular location at the cytoplasmic face of the plasma membrane [1]. Thus, the neutralization of p21Ras proteins in the cytoplasm using specific antibodies may block ras signalling and constitute a promising therapeutic strategy [2].

It is well known that whole antibodies can penetrate cells only with difficulty due to their large molecular size. In recent years, a series of low-molecular-weight antibodies containing antigen-binding domains have been explored to develop antibody-based drugs with better tumour penetration, such as antigen-binding fragment [3], single chain fragment variable (scFv) [4], and single-domain antibodies [5]. It has been found that scFv antibodies penetrate the cell membrane better than whole antibodies [6, 7] and result in no immunological rejections due to lacking the Fc fragment [8, 9], giving them advantages as intracellular immunization and therapeutic antibodies. Currently, scFv antibodies have been applied in many fields, including anti-viral and cancer therapy [10–12].

Both overexpression and mutation can activate *ras* genes. The overexpression of p21Ras has been detected in many human tumours [13–17]. The overexpression of *ras* family members led to the acquired resistance of cancer to cetuximab treatment [18]. It has been found that *ras* mutations are present in approximately 33 % of all human tumours [19]. *K-ras* mutations occur frequently in non-small-cell lung, colorectal, and pancreatic carcinomas; *H-ras* mutations are common in bladder, kidney, and thyroid carcinomas; and *N-ras* mutations are found in melanoma, hepatocellular carcinoma, and haematologic malignancies [20]. However, previously reported anti-p21Ras antibodies were derived from mutated p21Ras antigen [21–23]. In this study, we isolated hybridoma cell lines producing anti-p21Ras monoclonal antibodies, using wildtype p21Ras proteins as immunogens, prepared anti-p21Ras scFv antibodies from the hybridomas, and then investigated their immunoreactivity with human tumour cell lines and primary tumour tissues.

## Methods

### Preparation of the wildtype p21Ras proteins

The coding sequences (CDS) of the *H-ras*, *K-ras*, and *N-ras* genes were chemically synthetized according to their wildtype mRNA sequences published in NCBI GenBank: NM_005343 for *H-ras*, M54968 for *K-ras*, and BC005219 for *N-ras*. The restriction enzyme *Bam* HI cutting site GGATCC was ligated at the 5′ end of the CDS, and the *Hind* III cutting site AAGCTT was ligated at the 3′ end during synthesis. After digestion with *Bam* HI and *Hind* III, the three CDS fragments were ligated separately into the vector pET-28a(+) by T4 ligase. Then, recombinant expressing plasmids were transformed into *E. coli* BL21(DE3) and screened by kanamycin, induced by IPTG for p21Ras expression [24]. Expressed p21Ras proteins were purified by Ni^2+^-NTA resin with the mild denaturant urea and then underwent SDS-PAGE analysis, followed by dialysis for renaturation.

### Preparation of hybridomas producing broad-spectrum anti-p21 Ras mAb

Balb/c mice were immunized by injection with wildtype H-p21Ras expressed prokaryotically. The mouse splenic B lymphocytes were fused with myeloma cell lines SP2/0. After selective culture using HAT selective culture medium, the fused hybridoma cells were screened by an indirect ELISA method with all three wildtype p21Ras proteins and then cloned and subcloned to obtain hybridoma cell lines producing monoclonal antibodies against wildtype H-p21Ras, K-p21Ras and N-p21Ras. All hybridoma cell lines were subcloned twice. Finally, the hybridoma cell lines were injected into the peritoneal cavity of Balb/c mice to produce monoclonal antibodies [21]. A completed ARRIVE guidelines checklist is included in Additional file 1. Human cancer cell lines were used to identify the immunoreactivity of the monoclonal antibodies by Western blot. Primary tumour tissues and their corresponding normal tissues were employed to investigate the tumour reactivities of the monoclonal antibodies by immunohistochemical staining, and the results were described by HSCOREs [25].

### Construction of scFv phage display library

Hybridoma cell line KGH-R1, which produced excellent broad-spectrum anti-p21Ras mAb, was used to construct a phage scFv display library. Total RNA was isolated from KGH-R1 cells using RNAiso plus (MrcGene) and reverse transcribed to cDNA using the RevertAid™ H Minus First Strand cDNA Synthesis Kit (Fermentas). The heavy chain variable region (V_H_) and light chain variable region (V_L_) were amplified using primers provided by the Recombinant Phage Antibody System (GE Healthcare, RPAS). A DNA linker complementary to the 3′ end of V_H_ and 5′ end of V_L_ was ligated between the V_H_ and V_L_ fragments by overlapping extended PCR amplification to construct the scFv gene.

The phage scFv display library was constructed according to the RPAS system manual (GE Healthcare). Briefly, the scFv fragment genes and the phagemid pCANTAB-5E were sequentially digested with the *Sfi* I and *Not* I restriction enzymes. After purification by agarose gel extraction, both the scFvs fragments and the pCANTAB-5E vector with *Sfi* I and *Not* were ligated using T4 DNA ligase, transformed into competent *E. coli* TG1 cells, and cultured on SOBAG plates (SOB-AG, containing 100 μg/ml ampicillin and 2 % glucose) at 37 °C. All of the bacterial single colonies that grew on the SOBAG plate were collected, mixed and cultured in 2 × YTAG medium. The helper phage M13K07 at 10^11^ pfu was added to the collections and co-cultured at 30 °C for 1 h to obtain recombinant phages expressing the scFv antibodies and g3p fusion proteins on the surface. The scFv phages were purified and concentrated by polyethylene glycol (20 % PEG8000, 2.5 mol/L NaCl). The pellet was resuspended in PBS to obtain the phage scFv display library.

### Enrichment of scFv phage

The phage scFv display library underwent three rounds of panning using H-p21Ras, K-p21Ras and N-p21Ras protein antigens, respectively. Briefly, the flask was coated with p21Ras protein dissolved in 0.1 mM NaHCO_3_. After blocking with BSA, the library was added to the flask and incubated at 37 °C for 2 h, followed by washing with PBST (containing 0.05 % tween-20, pH 7.4) 10 times, followed by washing PBS (without Tween-20) an additional 10 times to remove unbound phage particles. Specifically bound phages were eluted with 0.1 M HCL/glycine (containing 0.1 % BSA, pH 2.2). The eluent was neutralized with pH 9.0 Tris/HCl, was used to infect TG1 cells and was then rescued with helper phage M13K07. These enriched scFv phages were used in the next round of panning [26, 27]. In each panning round, the numbers of input and output phages were counted using the double agar layer method.

### Identification of scFv-phages

*E. coli* TG1 cells were infected by the final enriched phage pools and cultured on an SOB-AG plate. Single colonies were picked and rescued individually with M13K07 in 96-well plates. The phages from individual wells were assayed by ELISA to detect their specific antigen binding activities, utilizing p21Ras proteins as antigens, scFv-phage as primary antibody, and HRP-conjugated mouse anti-M13 mAb as second antibody. The positive phages were further identified by *Sfi* I and *Not* I enzyme digestion and PCR amplification with pCANTAB-5E primers (forward, S1: 5-CAACGTGAAAAAATTATTATTCGC-3; reverse, S6: 5-GTAAATGAATTTTC TGTATGAGG-3). The amplified products were ligated into a pMD18-T vector for sequence analysis.

### Bioinformatics analysis of scFv gene and 3D modelling

The sequence of the scFv gene was blasted with known murine genes for homology analysis in the GenBank database, as described previously [28]. The amino acid residues, CDRs and FRs were determined according to the IMGT numbering system and Kabat by IgBLAST [28]. The 3D structure of scFv antibody was generated by SWISS-MODEL [29, 30].

### Expression of soluble scFv antibodies

The expression of soluble scFv antibodies was performed according to the manual and reference [31]. Briefly, the p21Ras-positve phages were used to infect HB2151 cells and cultured in plates. A single colony was picked and grown overnight to express soluble anti-p21Ras scFv antibodies with E-tag peptide at the end through the induction of IPTG. The soluble scFv antibodies were extracted from the HB2151 culture and concentrated by PEG8000/NaCl.

### Immunocytochemistry/immunohistochemistry and Western blot

Human tumour cell lines harbouring wildtype or mutated Ras [32, 33], primary tumour tissues and the corresponding normal tissues were employed to detect the immunoreactivities of the scFv antibodies by immunohistochemical staining. The soluble scFv antibodies were used as the primary antibody, whereas Anti-E-tag monoclonal antibody conjugated with HRP served as the second antibody to bind E-tag protein ligated to the end of the scFv antibody. The scFv immunoreactivity with p21Ras was further detected by Western blot in the tumour cell lines MDA-MB-435, MDA-MB-231 and SKOV3 (ATCC USA) and the normal cell line KMB17 (normal human embryonic diploid lung fibroblast cell, constructed by the Institute of Medical Biology, Chinese Academy of Medical Sciences). β-actin was used as a control, with anti-β-actin monoclonal antibody conjugated with HRP.

### Ethics statement

This study including animal work has the approval of the Ethics Board of Kunming General Hospital and is also in accordance with the Helsinki Declaration of 1975. Written informed consent was obtained from every patient. All human tissues were processed anonymously.

## Results

### Preparation of hybridomas and anti-p21Ras monoclonal antibodies

**Fig. 1:**
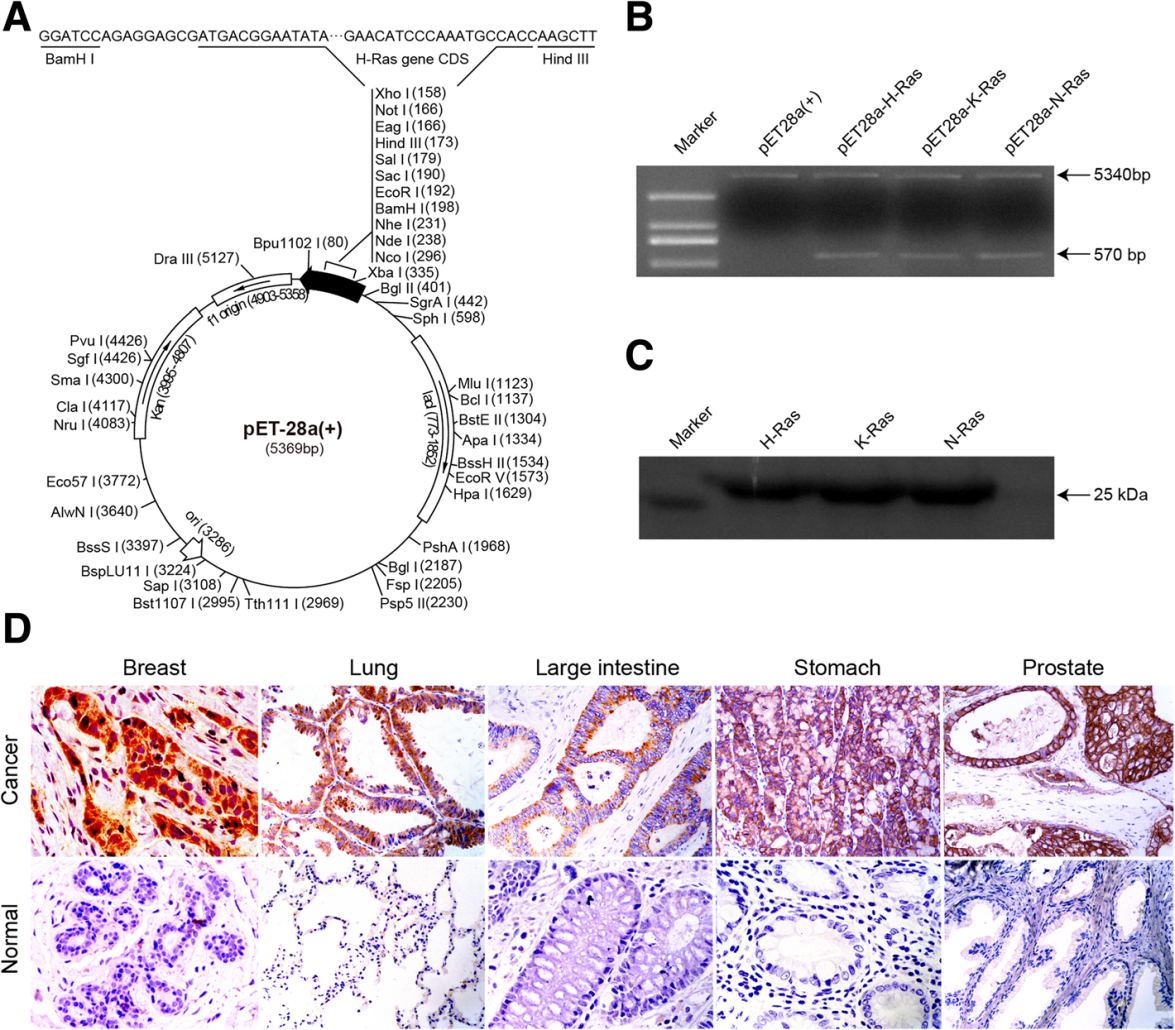
Wildtype p21Ras proteins and anti-p21Ras monoclonal antibodies. **a** The plasmid map of recombinant pET-28a(+) vector expressing *H-ras*, in which *H-ras* gene CDS with *Bam* HI and *Hind* III restriction enzyme sites was cloned into a pET-28a(+) vector with a histidine tag-compatible end. **b** The three recombinant pET28a(+) plasmids were digested into two fragments by *Bam* HI and *Hind* III, the 570 bp of the *ras* gene CDS and 5340 bp of the pET-28a(+) vector. **c** SDS–PAGE analysis showed that the molecular weight of the three p21Ras proteins with His tag was 25 kDa. **d** KGH-R1 monoclonal antibody against three p21Ras proteins demonstrated strong immunoreactivity to cancer tissues but negative immunoreactivity to corresponding normal tissues

By cloning the CDS of the *H-ras*, *K-ras*, *N-ras* genes into pET-28(+) vectors, we constructed a prokaryotically expressive vector (Fig. 1a, b) that effectively expressed H-p21Ras, K-p21Ras and N-p21Ras protein in *E. coli* BL21. SDS-PAGE analysis showed that the apparent molecular weight of the three p21Ras proteins was 25 KD (Fig. 1c). The purity of p21Ras protein was more than 95 %.

H-p21Ras was used as an immunogen to immunize BALB/c mice to isolate hybridomas. To achieve monoclonal antibodies that simultaneously recognized H-p21Ras, K-p21Ras and N-p21Ras, the three p21Ras were used as antigens to screen the hybridomas in an ELISA assay. In total, we obtained 13 hybridomas that produced monoclonal antibodies against the three different p21Ras proteins. Karyotype analysis showed that the median chromosome numbers of the 13 hybridomas were 97–100. Immunochromatography indicated that the monoclonal antibodies produced by all 13 hybridomas were of the IgG2b/κ subtype. Monoclonal antibodies in ascites reached a high titre of 1:128000. The immunoreactivities of the monoclonal antibody KGH-R1 were determined by Western blot and immunohistochemistry. The KGH-R1 monoclonal antibody demonstrated positive immunoreactivities in all 13 human solid tumour cell lines and 2/6 leukaemia cell lines (Table 1). In all 14 types of primary human cancer tissues tested here, the monoclonal antibody presented strong immunoreactivity but showed weak or negative immunoreactivity in the corresponding normal tissues (Table 2, Fig. 1d).

**Table 1:** Immunoreactivity of KGH-R1 mAb in human tumour cell lines

Tumour cell lines^a^	Ras status^b^	Western blot	Immunocytochemistry
QGY7703	Unknown	+	+
SMMC7721	Unknown	+	+
HepG2	N, mutant	+	+
BGC823	Unknown	+	+
MKN28	Unknown	+	+
HCT116	K, mutant	+	+
T24	H, mutant	+	+
SKOV3	Unknown	+	+
MDA-MB-231	K, mutant	+	+
MDA-MB-435	Unknown	+	+
MCF7	Unknown	+	+
HeLa	Wildtype	+	+
Hep2	Unknown	+	+
C8166	Unknown	+	+
K562	Unknown	+	+
Daud I	Unknown	-	-
HL60	N, mutant	-	-
MT4	Unknown	-	-
THP1	N, mutant	-	-

^a^QGY7703, SMMC7721, HepG2: hepatocarcinoma; BGC853, MKN28: gastric cancer; HCT116: colorectal cancer; T24: bladder cancer; SKOV3: ovary cancer; MDA-MB-231, MDA-MB-435, MCF7: breast cancer; HeLa: cervical cancer; Hep2: laryngocarcinoma; C8166, K562, Daud I, HL60, MT4, THP1: leukaemia. ^b^published data

**Table 2:** Immunoreactivity of KGH-R1 mAb in human tumours and corresponding normal tissues

Tumour	Cases	Immunoreactivity (HSCOREs^a^)	
		Primary tumour tissues	Normal tissues
Colorectal cancer	30	208.69 ± 84.40	6.88 ± 1.53
Gastric cancer	30	155.29 ± 87.50	7.86 ± 2.79
Oesophageal cancer	30	163.73 ± 66.00	8.50 ± 3.28
Bladder cancer	30	146.76 ± 95.36	8.10 ± 2.43
Lung adenocarcinoma	30	238.63 ± 72.00	7.00 ± 1.77
Lung squamous cell carcinoma	30	178.26 ± 83.99	7.00 ± 1.77
Lung small cell carcinoma	30	134.38 ± 91.58	7.00 ± 1.77
Papillary thyroid carcinoma	30	239.33 ± 75.51	8.43 ± 2.32
Hepatocarcinoma	30	184.38 ± 96.75	11.29 ± 5.51
Prostate carcinoma	30	114.77 ± 70.59	13.00 ± 4.24
Breast cancer	30	197.46 ± 56.33	9.23 ± 3.19
Endometrial cancer	30	139.29 ± 86.00	15.75 ± 5.72
Glioma	30	97.60 ± 8.85	17.30 ± 8.32
Renal cell carcinoma	30	117.50 ± 73.86	16.00 ± 5.85

^a^Mean ± SD

### Construction and panning of scFv phage display library

**Fig. 2:**
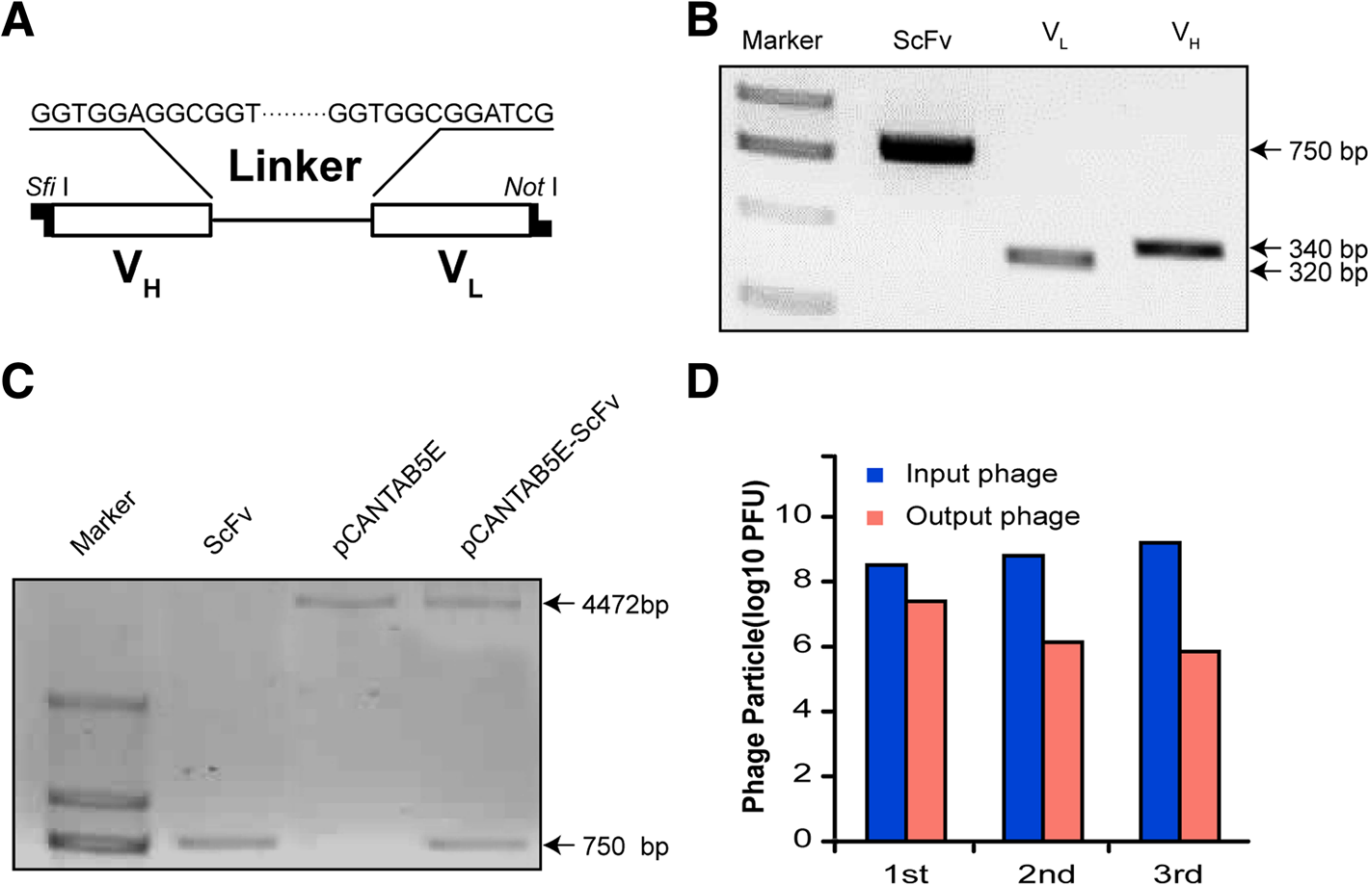
Preparation of anti p21Ras scFv. **a** Schematic diagram of scFv. The variable region of the heavy chain (V_H_) and the variable region of the light chain (V_L_) were combined with a polypeptide linker. **b** Construction of scFv gene. The 340 bp V_H_ fragment and 325 bp V_L_ fragment were amplified from the cDNA of the KGH-R1 hybridoma. This assembly reaction ultimately produces 750 bp of scFv. **c** The recombinant pCANTAB5E-scFv plasmid was digested into two fragments by *Sfi* I *and Not* I: 750 bp of scFv and 4472 bp of pCANTAB-5E vector. **d** The phage-ELISA showed that after three rounds of panning by H-p21Ras, K-p21Ras and N-p21Ras antigens, in turn, the number of output phages (unbound phages) was constant at 1 × 10^6^ PFU/ml, whereas the number of input phage was constant at 1 × 10^9^ PFU/ml

The scFv gene consists of a heavy chain variable region (V_H_) and a light chain variable region (V_L_), joined by a neutral linker (Fig. 2a). We amplified V_H_ (340 bp) and V_L_ (320 bp) from the cDNA of the KGH-R1 hybridoma. Then, we connected both to DNA linker by overlapping extended PCR to construct a 750 bp scFv gene (Fig. 2b). The scFv gene repertoire was ligated to the phagemid pCANTAB 5E and then transformed into competent TG1 cells and grown in SOBAG plates, obtaining 1 × 10^7^ TG1 colonies transformed by the recombinant phage plasmids (Fig. 2c). We collected all of the TG1 colonies, co-cultured them with M13KO7 helper phages, and established the scFv phage display library, in which scFv-g3p fusion proteins were expressed at the tips of the phages. To obtain phages expressing anti-p21Ras scFv antibodies from the library, the library was selected and panned using the H-p21Ras, K-p21Ras, and N-p21Ras proteins, in turn. After three rounds of panning, the eluted phages were maintained at 1 × 10^6^ pFU/mL, and the number of input phage pools was maintained at 1 × 10^9^ pFU/mL (Fig. 2d).

### Characteristics of anti-p21Ras scFv antibodies

**Fig. 3:**
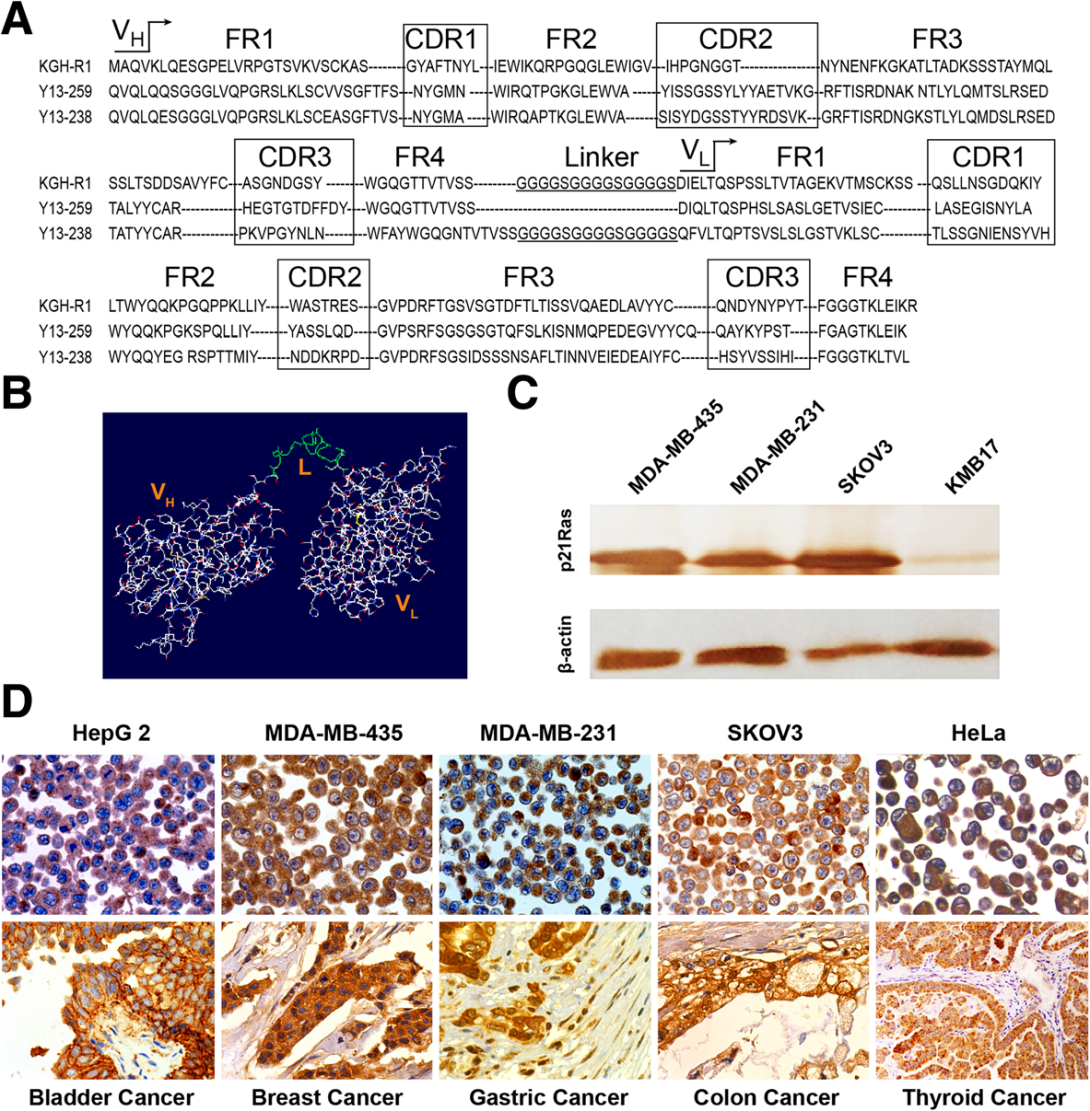
Bioinformatics and immunoreactivity of the anti-p21Ras scFv. **a** Amino acid sequence of anti-p21Ras scFv with appropriate regions for framework and CDR residues, differing from the previously reported Y13-159 scFv and Y13-238 scFv. **b** 3D model of anti-p21Ras scFv generated by bioinformatics. Variable heavy chain (V_H_) and light chain (V_L_) regions of the antibody were connected by a single 15-amino acid linker (L, green). **c** Western blot detected anti-p21Ras scFv antibody-specific binding with p21Ras protein in the cells, where strong immunoreactivity was found in p21Ras-overexpressing tumour cell lines; however, weak immunoreactivity was observed in the normal cell line KMB17 with low p21Ras expression. **d** Immunohistochemistry revealed that anti-p21Ras scFv antibody exhibited strongly positive staining in human tumour cell lines and primary solid tumour tissues

To obtain monoclonal phages expressing anti-p21Ras scFv antibodies, we infected TG1 cells with the enriched scFv phage pool and plated them on SOBAG plates. Then, 40 colonies were rescued by M13K07. Indirect ELISA tests showed that all 40 colonies expressed scFv antibodies that could bind H-p21Ras, K-p21Ras and N-p21Ras simultaneously. Recombinant phagemids were isolated from each of the 40 colonies and digested by *Sfi* I and *Not* I and then ligated with pMD18-T vector for DNA sequencing. Sequence analysis demonstrated that the sequences of the 40 colonies were the same. The number of amino acids and complementary determining regions (CDRs) of the V_H_ and V_L_ domains were determined using IgBLAST. KGHR1-scFv had 738 nucleotides encoding 246 amino acids, of which V_H_ consisted of 117 amino acids, V_L_ consisted of 114 amino acids, and the flexible amino acid linker consisted of 15 amino acids. Both the V_H_ and V_L_ fragments had 3 CDR regions and 4 FR regions (Fig 3a). Homology analysis revealed that V_H_ belongs to murine subfamily III and that V_L_ belongs to the subfamily VII κ chains. We constructed a three-dimensional model of the scFv antibody by the SWISS MODEL method. In the 3D model, scFv consists of the V_H_ chain and the V_L_ chain connected by a polypeptide linker chain (Fig. 3b).

### Immunoreactivity of soluble anti-p21Ras scFv antibodies

Soluble anti-p21Ras scFv antibodies were prepared by infecting the non-amber suppressor *E. coli* strain HB2151 with the recombinant phages. The Western blot assay showed that anti-p21Ras scFv antibody could specifically bind the p21Ras protein in the tested cells (Fig. 3c). We further evaluated the immunoreactivities of scFv in human tumour cell lines, primary solid tumour tissues and normal tissues by immunohistochemical staining. The results demonstrated that the soluble scFv displayed strong immunostaining in all the human tumour cell lines and all types of primary human cancer tissues tested here. However, all of the corresponding normal tissues showed negative immunostaining. The granular positive signal was mainly located in the cytoplasm and membrane (Table 3, Fig. 3d). The results indicated that the obtained scFv could recognize the p21Ras antigen epitope in tumour cells.

**Table 3:** Immunoreactivity of KGHR1-scFv antibody in human tumour tissues and cell lines

Immunoreactivity (HSCOREs^a^)				Immunocytochemistry	
Tumour tissues	Cases	Primary tumour tissues	Normal tissues	Tumour cell lines	Results
Colorectal cancer	30	180.54 ± 79.21	7.31 ± 2.13	BGC853	+
Gastric cancer	25	150.17 ± 64.31	7.13 ± 1.99	MKN28	+
Oesophageal cancer	25	151.42 ± 59.13	8.21 ± 2.38	HCT116	+
Bladder cancer	25	149.37 ± 79.27	7.23 ± 2.15	QGY7703	+
Lung adenocarcinoma	20	199.45 ± 69.11	8.13 ± 2.17	SMMC-7721	+
Lung squamous cell carcinoma	20	185.37 ± 71.64	7.53 ± 2.23	HepG2	+
Lung small cell carcinoma	19	146.25 ± 83.00	7.78 ± 2.17	MDA-MB-231	+
Papillary thyroid carcinoma	20	191.67 ± 82.71	7.93 ± 2.76	MDA-MB-435	+
Hepatocarcinoma	22	168.25 ± 79.33	8.47 ± 3.18	MCF7	+
Prostate carcinoma	25	145.37 ± 75.35	8.52 ± 3.43	HeLa	+
Breast cancer	24	165.73 ± 63.28	7.93 ± 2.56	SKOV3	+
Endometrial cancer	30	153.32 ± 57.00	8.73 ± 3.63	Hep2	+
Glioma	20	131.50 ± 42.59	10.28 ± 5.00	T24	+
Renal cell carcinoma	24	89.61 ± 53.73	11.27 ± 4.91		

^a^Mean ± SD

## Discussion

Antibody-based drugs are promising agents for cancer therapy. In recent years, certain antibody drugs have been clinically approved and used frequently in cancer treatment. Trastuzumab, a chimeric monoclonal antibody against Her2 protein, is the most impressive target drug for breast cancer therapy [34, 35]. Rituximab, an anti-CD20 monoclonal antibody, is another of the most effective drugs in B cell malignant lymphoma treatment [36]. The gene *ras* is a well-known and important oncogene involved in the development and progression of many human tumours, but no antibody drugs targeting the *ras* gene have been applied clinically. The main reason is that the intracellular location of p21Ras has limited the effects of antibodies because intact antibodies can not penetrate the cell membrane. Thus, small molecular antibodies, such as scFv, are proposed to treat *ras*-derived tumours.

Because hybridoma cell lines are the most important resources for scFv [37–39], we established anti-p21Ras hybridomas using prokaryotically expressed wildtype H-p21Ras protein as an immunogen. H-p21Ras, K-p21Ras and N-p21Ras were used to screen the hybridoma clones. Finally, the hybridoma KGH-R1, producing a broad-spectrum monoclonal antibody against the three p21Ras proteins, was isolated. Interestingly, the monoclonal antibodies reacted with both wildtype and mutated p21Ras in human tumour cell lines and presented excellent immunoreactivities with the majority of human tumour cell lines and primary tumour tissues but negative immunostaining in the corresponding normal tissues. The characteristics mentioned above give these antibodies important value as therapeutic prodrugs. To our knowledge, this study is the first to use wildtype p21 Ras as an immunogen to prepare monoclonal antibodies. The anti-p21Ras monoclonal antibodies reported previously were derived from mutated p21Ras, of which Y13-259 and Y13-238 were generated using p21 protein encoded by the *v-ras* gene of the Harvey murine sarcoma virus as an immunogen [22]. RAP-1 ~ 5 were generated utilizing a synthetic H-p21Ras peptide reflecting amino acid positions 10–17 with a mutation at codon 12 as the immunogen [23]. RASK1 ~ 16 were generated using mutated K-p21Ras from Kirsten murine sarcoma virus as the immunogen [21].

Antibody genes can be fused into phage genes, and then the antibodies can be expressed and displayed on the surface of the phages as fusion proteins [40]. The phage display library has thus become the most popular technique for constructing and selecting scFv antibodies from hybridomas or B lymphocytes due to its benefits, such as the high diversity of the antigenic repertoire and the ability to rapidly select specific antibodies [41]. In this study, we employed the phage display library method to isolate scFv antibodies against human p21Ras proteins. Fortunately, we obtained scFv antibodies that maintained all of the immunoreactivities of the original monoclonal KGH-R1 antibodies, including the recognition of wildtype and mutated p21Ras proteins, reaction with H-p21Ras, K-p21Ras and N-p21Ras, and strong immunostaining in tumour cell lines and primary tumours. These results showed that scFv is useful for ras signalling blockage and may be a therapeutic antibody for *ras*-derived tumours.

The scFv antibodies presented here were dramatically different from previous anti-p21Ras scFv in both their nucleotide sequences and their amino acid sequences [42, 43]. There was a 49.8 % difference from Y13-259 scFv and a 53.73 % difference from Y13-238 scFv, mostly located in the CDRs (Fig. 3a). Our data indicated that KGH-R1 scFv recognizes different antigen epitopes, based on a comparison of Y13-259 scFv and Y13-238 scFv, and it is a novel anti-p21Ras scFv antibody.

## Conclusions

In conclusion, this study presents a novel anti-p21Ras scFv antibody, KGH-R1 scFv, produced by hybridoma and phage scFv display library methods. Sequence analysis showed that KGH-R1 scFv antibody has an approximately 50 % difference from reported anti-p21Ras scFv antibodies. The KGH-R1 scFv antibody could recognize wildtype and mutated H-p21Ras, K-p21Ras, and N-p21Ras and exhibited strong immunoreactivity with human tumour cell lines and primary tumours. It is suggested that the scFv may be useful for *ras* signalling blockage and as a therapeutic antibody for *ras*-derived tumours.

## Additional files

Additional file 1:
**The ARRIVE Guidelines Checklist.** (DOC 694 kb)

## Abbreviations

scFv: Single chain fragment variable
kD: KiloDalton
GTP: Guanosine triphosphate
CDS: Coding sequences
NCBI: National Center for Biotechnology Information
IPTG: Isopropyl β-D-1-thiogalactopyranoside
SDS-PAGE: Sodium dodecyl sulfate-polyacrylamide gel electrophoresis
mAb: Monoclonal antibody
HAT: Hypoxanthine, aminopterin, thymidine
ELISA: Enzyme-linked immunosorbent assay
V_H_: Heavy chain variable region
V_L_: Light chain variable region
PBS: Phosphate buffered saline
PBST: PBS containing 0.05 % tween-20
BSA: Albumin from bovine serum
HRP: Horseradish peroxidase
CDR: Complementarity-determining region
FR: Framework region
Her2: Human epidermal growth factor receptor 2
