# Supplementary material for: A novel anti-p21Ras scFv antibody reacting specifically with human tumour cell lines and primary tumour tissues
Source: BMC Cancer. 2016 Feb 20;16:131. doi: 10.1186/s12885-016-2168-6 (PMC4761205; doi:10.1186/s12885-016-2168-6)

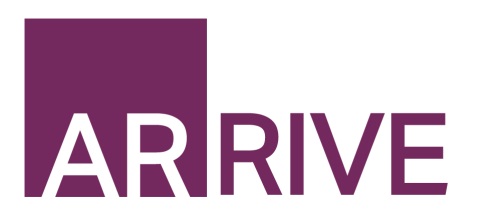


The ARRIVE Guidelines Checklist

Animal Research: Reporting In Vivo Experiments

Carol Kilkenny^1^, William J Browne^2^, Innes C Cuthill^3^, Michael Emerson^4^ and Douglas G Altman^5^

*^1^The National Centre for the Replacement, Refinement and Reduction of Animals in Research, London, UK, ^2^School of Veterinary Science, University of Bristol, Bristol, UK, ^3^School of Biological Sciences, University of Bristol, Bristol, UK, ^4^National Heart and Lung Institute, Imperial College London, UK, ^5^Centre for Statistics in Medicine, University of Oxford, Oxford, UK.*

|  | | ITEM | RECOMMENDATION | Section/ Paragraph |
| --- | --- | --- | --- | --- |
|  | 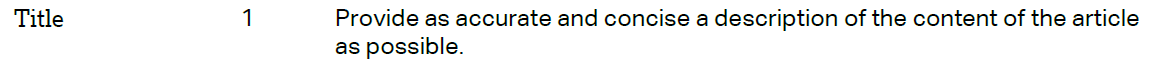 | | | title |
|  | 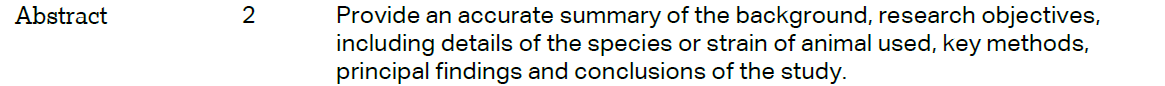 | | | abstract |
|  | INTRODUCTION | | |  |
|  | 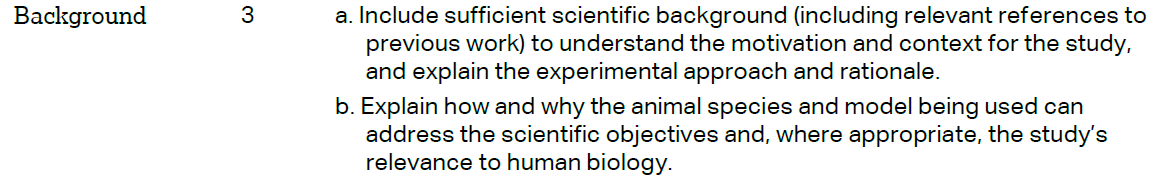 | | | Line 60-96 |
|  | 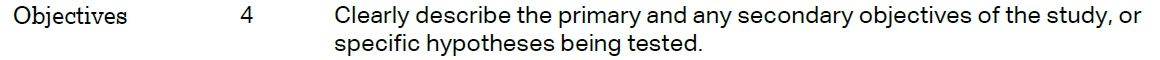 | | | Line 92-96 |
|  | METHODS | | |  |
|  | 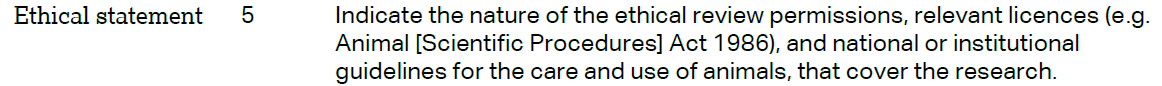 | | | Line 200-203 |
|  | 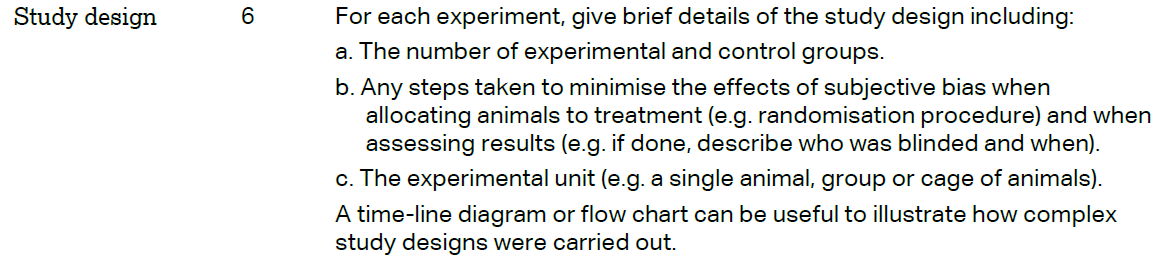 | | | Line 98-125 |
|  | 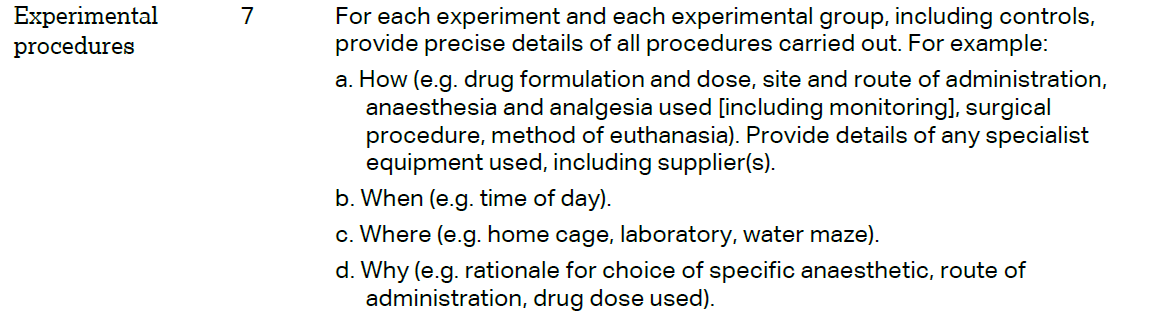 | | | Line 98-125 |
|  | 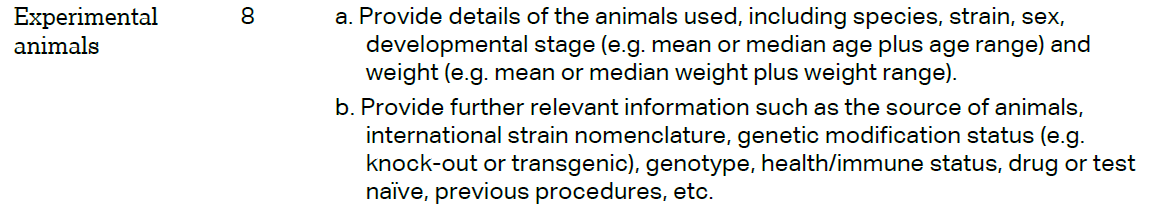 | | | Line 112 |

The ARRIVE guidelines. Originally published in *PLoS Biology*, June 2010^1^

|  | 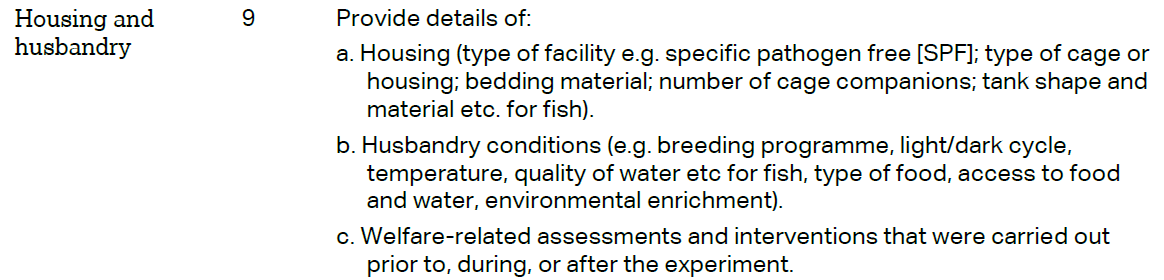 | Experimental animal room | |
| --- | --- | --- | --- |
|  | 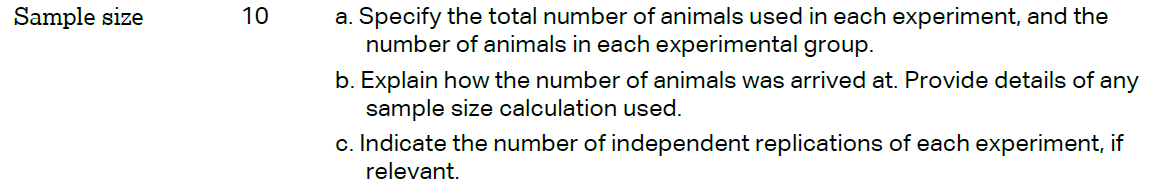 | five | |
|  | 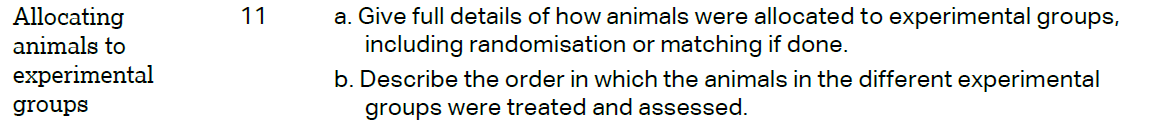 | none | |
|  | 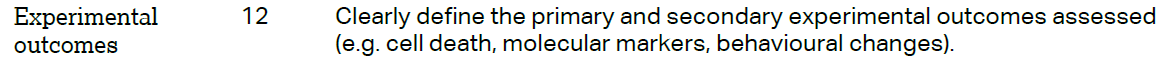 | Line 204-218 | |
|  | 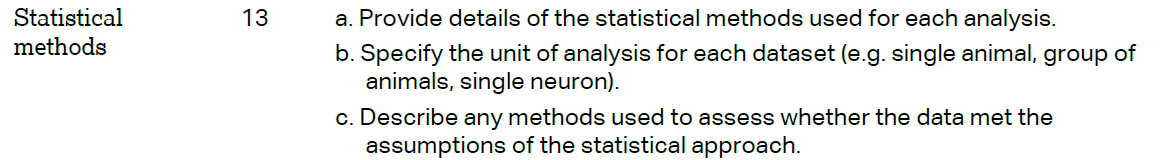 | none | |
|  | RESULTS |  | |
|  | 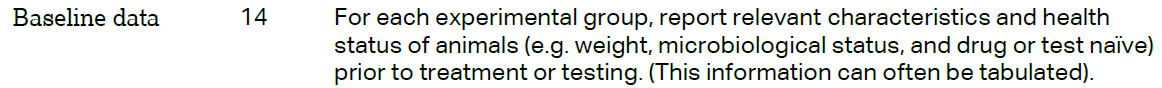 | Good condition | |
|  | 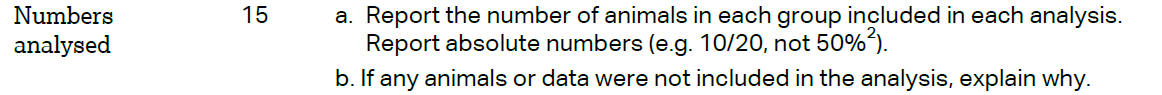 | 5/5 | |
|  | 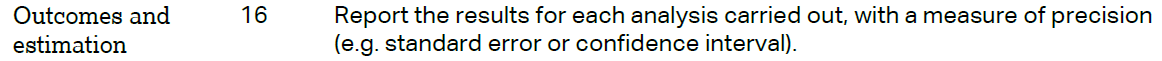 | Line 204-218 | |
|  | 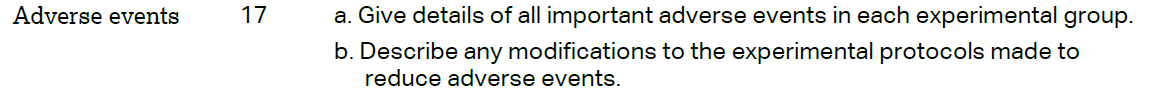 | none | |
|  | DISCUSSION |  | |
|  | 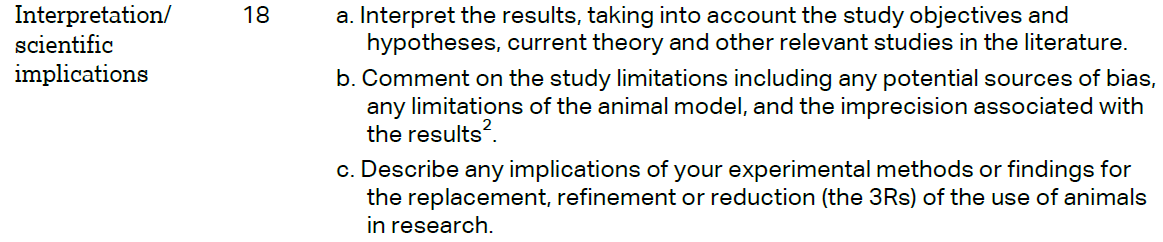 | Line 267-314 | |
|  | 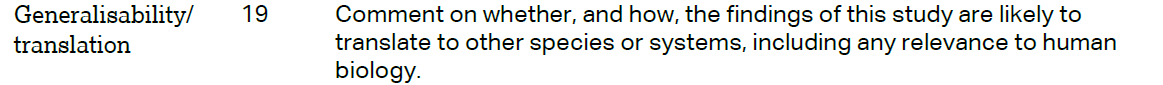 | Line 267-323 | |
| 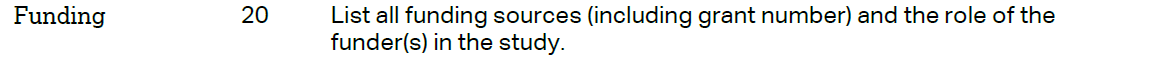 | | Line 349-351 |  |


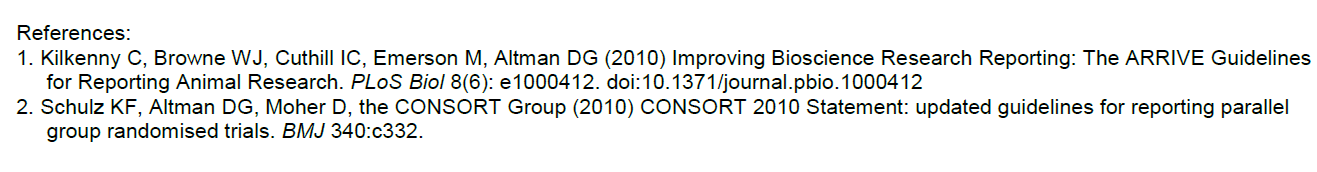

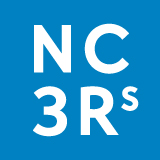

Supplement: Additional file 1: — The ARRIVE Guidelines Checklist. (DOC 694 kb) [file 12885_2016_2168_MOESM1_ESM.doc]
